# Analysis of host cell binding specificity mediated by the Tp0136 adhesin of the syphilis agent *Treponema pallidum* subsp. *pallidum*

**DOI:** 10.1371/journal.pntd.0007401

**Published:** 2019-05-09

**Authors:** Vitomir Djokic, Lorenzo Giacani, Nikhat Parveen

**Affiliations:** 1 Department of Microbiology, Biochemistry and Molecular Genetics, Rutgers New Jersey Medical School, Newark, New Jersey, United States of America; 2 Department of Medicine, Division of Allergy and Infectious Diseases, University of Washington, Seattle, Washington, United States of America; 3 Department of Global Health, University of Washington, Seattle, Washington, United States of America; UAMS, UNITED STATES

## Abstract

**Background:**

Syphilis affects approximately 11 million people each year globally, and is the third most prevalent sexually transmitted bacterial infection in the United States. Inability to independently culture and genetically manipulate *Treponema pallidum* subsp. *pallidum*, the causative agent of this disease, has hindered our understanding of the molecular mechanisms of syphilis pathogenesis. Here, we used the non-infectious and poorly adherent B314 strain of the Lyme disease-causing spirochete, *Borrelia burgdorferi*, to express two variants of a known fibronectin-binding adhesin, Tp0136, from *T*. *pallidum* SS14 and Nichols strains. Using this surrogate system, we investigated the ability of Tp0136 in facilitating differential binding to mammalian cell lines offering insight into the possible role of this virulence factor in colonization of specific tissues by *T*. *pallidum* during infection.

**Principal findings:**

Expression of Tp0136 could be detected on the surface of *B*. *burgdorferi* by indirect immunofluorescence assay using sera from a secondary syphilis patient that does not react with intact B314 spirochetes transformed with the empty vector. Increase in Tp0136-mediated adherence of B314 strain to human epithelial HEK293 cells was observed with comparable levels of binding exhibited by both Tp0136 alleles. Adherence of Tp0136-expressing B314 was highest to epithelial HEK293 and C6 glioma cells. Gain in binding of B314 strain expressing Tp0136 to purified fibronectin and poor binding of these spirochetes to the fibronectin-deficient cell line (HEp-2) indicated that Tp0136 interaction with this host receptor plays an important role in spirochetal attachment to mammalian cells. Furthermore, preincubation of these cell lines with fibronectin-binding peptide from *Staphylococcus aureus* FnbA-2 protein significantly inhibited binding of B314 expressing Tp0136.

**Conclusions:**

Our results show that Tp0136 facilitates differential level of binding to cell lines representing various host tissues, which highlights the importance of this protein in colonization of human organs by *T*. *pallidum* and resulting syphilis pathogenesis.

## Author summary

Syphilis is one of the most prevalent sexually transmitted infections that affect millions of people around the world. The causative bacterium, *Treponema pallidum* subsp. *pallidum*, can be transmitted from mother to fetus during maternal infection, resulting in adverse pregnancy outcomes. Although timely treatment of syphilis is highly effective, untreated infection causes late syphilis that affects virtually every organ and leads to serious clinical manifestations. Therefore, syphilis remains a serious healthcare problem. *T*. *pallidum* cannot be grown in laboratory using traditional methods, which has slowed the progress in understanding this pathogen biology and pathogenesis. We employed a novel approach of using a related bacterium, *Borrelia burgdorferi*, to express Tp0136 protein from two different *T*. *pallidum* isolates to study the function of this protein. This strategy enabled us to demonstrate the ability of this protein to bind to fibronectin and laminin receptors present on the surface of various host cells. We showed that Tp0136 facilitates binding to only those host cells that produce fibronectin. In addition, we found that Tp0136-mediated binding is not equivalent in all host cell types, suggesting that the protein could help in colonization of specific human organs and tissues during infection by *T*. *pallidum*.

## Introduction

Although syphilis can be easily diagnosed and treated, 11 million new infections are estimated to occur annually, contributing to a global prevalence of ~36 million cases [1–3]. Congenital syphilis resulting from vertical transmission of the syphilis agent, *Treponema pallidum* subsp. *pallidum* (*T*. *pallidum*), is also a significant global health issue because an estimated 1.4 million pregnant women acquire syphilis every year. Congenital syphilis is a major cause of fetal loss, stillbirth, or malformations in the newborns, particularly in low-income countries where most cases occur. Evidence that symptomatic syphilis infection increases the risk of HIV transmission and acquisition [4] further emphasizes the magnitude of the threat posed to public health by this organism, and the need to better understand the molecular basis of syphilis pathogenesis. A better understanding of *T*. *pallidum* pathogenesis could lead to development of new approaches to control the spread of this infection.

*T*. *pallidum* is a slow growing bacterium and is considered the most virulent among the species and subspecies that cause human treponematoses because it causes serious systemic disease [5]. Following infection, *T*. *pallidum* rapidly disseminates to distant tissues and organs via the circulatory and lymphatic system in the early stages of the disease [6, 7]. In addition to crossing the placenta to cause congenital infection, the syphilis spirochete is also capable of passing through the blood-brain barrier, an event that can lead to the early and late neurological manifestations of the disease. Although the sequence of the first *T*. *pallidum* genome greatly helped in the identification of potential virulence factors of this pathogen, progress in the understanding of the pathogenesis of syphilis is hindered by several limitations inherent to the study of this microorganism. Such limitations include the inability to grow *T*. *pallidum* continuously *in vitro* in pure culture, thus making it extremely difficult to genetically manipulate this pathogen. A newly described tissue culture approach, in which *T*. *pallidum* is co-cultured with Sf1Ep epidermal cells of cottontail rabbits [8], will likely open new possibilities for physiological studies that haven’t been feasible until now. Another limitation of studying *T*. *pallidum* is that its envelope is exceedingly fragile compared to more conventional Gram-negative organisms [9] due to the absence of lipopolysaccharide (LPS) in its outer membrane and a very thin peptidoglycan layer that is only loosely associated to the outer membrane. Furthermore, *T*. *pallidum* outer membrane displays a very low density of proteins on its surface [10–15]. Because genetic manipulation of *T*. *pallidum* is still not possible, investigators have started using heterologous expression systems to better characterize the virulence factors of this spirochete [16–19].

Attachment to host components is a crucial initial step in the pathogenesis of the extracellular pathogens like *T*. *pallidum* that facilitates establishment of infection and tissue colonization [20, 21]. Although *T*. *pallidum’s* ability to adhere to a variety of host cell types was known since the 70s [22], significant progress toward the identification and functional characterization of *T*. *pallidum* adhesins has materialized only in the last two decades [16, 19, 23–27]. These investigations led to identification of several *T*. *pallidum* adhesins; including laminin- (Tp0751) and fibronectin- (Tp0136, Tp0155, Tp0483) binding proteins. However, these studies mostly relied on the use of recombinant soluble versions of these proteins to determine their receptors and role in adhesion, an approach that does not account for the conformational differences from their natural, outer membrane-associated counterpart and might also lack post-translational modifications. To bypass this obstacle, surrogate systems have been adapted in several recent studies to better define the role of adhesins. Thus, surrogate systems were used to investigate the laminin-binding lipoprotein Tp0751 [17], and *T*. *pallidum* lipoprotein Tp0435 that facilitates host cell binding [16], although the cellular receptor for Tp0435 has yet to be identified. Both of these studies employed the causative agent of Lyme disease, *Borrelia burgdorferi* (*B*. *burgdorferi*), as a surrogate spirochete to enable gain-of-function approach. *B*. *burgdorferi* was found to be an excellent heterologous system to examine localization of surface proteins and to study functions of *T*. *pallidum* lipoproteins. *T*. *pallidum* and *B*. *burgdorferi* are structurally and physiologically related spirochetes and they are suggested to process and present lipoproteins in a similar manner [28, 29]. It is therefore expected that processing of the translated Tp0136 protein facilitated by *B*. *burgdorferi* type II signal peptidase is followed by lipidation of the invariant cysteine residue in the lipobox, which becomes the first amino acid of the mature lipoprotein. Furthermore, *B*. *burgdorferi* is particularly useful to study *T*. *pallidum* adhesins because non-pathogenic and poorly adherent derivatives of this spirochete have been obtained by long-term *in vitro* cultivation of the wild-type strains [16, 30, 31]. Additionally, expression of the native *T*. *pallidum* genes in a related organism served as an alternative way to corroborate surface-exposure of these proteins, whose localization on the surface of *T*. *pallidum* is notoriously difficult.

In this study, we used the poorly adherent *B*. *burgdorferi* B314 strain to further characterize the role of *T*. *pallidum* Tp0136 adhesin. The role of this lipoprotein in fibronectin binding was first demonstrated by Brinkman *et al*. [32], and subsequently confirmed by Ke and coworkers [27], who evaluated the binding activity of different isoforms of Tp0136, known to be heterogeneous among *T*. *pallidum* isolates, to both tissue and plasma fibronectin. They concluded that this protein preferentially binds the tissue form of fibronectin, irrespective of the Tp0136 variant tested in those experiments. Here we used the *B*. *burgdorferi* B314 strain to: (a) express two allelic variants of Tp0136, (b) investigate the role of this protein in mediating adhesion to various primate cell lines by attachment to fibronectin and potentially laminin, and (c) evaluate Tp0136 likely contribution to *T*. *pallidum* colonization of different tissues.

## Materials and methods

### Ethics statement

All mouse experiments were performed in accordance with the provisions of the Animal Welfare Act, the Guide for the Care and Use of Laboratory Animals, and the PHS Policy on Humane Care and Use of Laboratory Animals. Experiments were conducted under the protocol # 14011D0617 approved by the Rutgers Biomedical and Health Sciences IACUC.

### Cloning of *tp0136* gene for expression of recombinant Tp0136 and for transformation of *B*. *burgdorferi*

High passage *B*. *burgdorferi* strain B314 [33, 34] was grown in BSKII medium containing 6% rabbit serum at 33°C. Genomic DNA from the *T*. *pallidum* SS14 and Seattle-Nichols strains were used to amplify the *tp0136* gene as previously reported [27] using the Sense: (5’- GGGGTACCTCTATTACGAGAAGGAGCGGC) and Antisense: (5’- AGAGTCGACGCAGACAAAACCCTCACGATT) primers with KpnI and SalI sites underlined. PCR products were cloned into the TOPO-XL vector (Invitrogen) according to the manufacturer’s protocol. The amplicon included 686 nucleotides upstream of the Tp0136 ORF, containing the gene putative promoter, and 460 nucleotides from the gene 3’-flanking region. After sequence confirmation (S1 Fig), the insert was sub-cloned using KpnI and SalI digestion into a *B*. *burgdorferi* shuttle vector pJSB175, encoding a codon-optimized firefly luciferase, and the plasmid was used for transformation and plating of *B*. *burgdorferi* B314 strain, as previously described [16, 35, 36]. For expression of recombinant Tp0136, the *tp0136* ORF without the first 48 nucleotides encoding the first 16 amino acids was amplified using the primers; 5Tp0136-sol (5’- GGGGGATCCATGACGGTGGTGCGCGCGGT) with BamH1, and 3Tp0136-sol (5’- GGGAAGCTTTTACTCGCGGTTCCAGGAGCACGT) with HindIII sites underlined and cloned in pET30a vector containing a 6xHis tag at the amino-terminal end of the recombinant protein to allow purification by affinity chromatography.

### Expression and purification of recombinant Tp0136 from *E*. *coli* and generation of anti-Tp0136 mouse antibodies

Polyhistidine-tagged Tp0136 (pET30a-*tp0136*) was expressed in *E*. *coli* BL21(pLysS) strain and recombinant Tp0136 from Nichols strain purified with the Nickel-affinity purification kit. Antibodies were raised against purified Tp0136 in female BALB/c mice using our previously described protocol [37].

### Expression of Tp0136 on the surface of *B*. *burgdorferi* B314 determined by indirect Immunofluorescence assay (IFA)

For examination of protein expression in *B*. *burgdorferi* strains, about 1-2x 10^8^ cells/ml were centrifuged and washed three times with PBS containing 0.2% bovine serum albumin (PBS/BSA) to remove growth medium components. IFA was conducted using bacteria centrifuged over coverslips placed in 24-well plate as described previously [37]. Briefly, after fixation with 3% paraformaldehyde in PBS for 1h and blocking with PBS/5% BSA/5% heat-inactivated goat serum at room temperature for 1h, a secondary syphilis patient serum (1:100 dilution), previously obtained from Dr. Arturo Centurion-Lara at the University of Washington, or anti-Tp0136 (Nichols strain) mouse serum (also used at 1:100) was added and plate incubated for 1h at room temperature. Coverslips were washed three times with PBS for five minutes each at room temperature, and subsequently incubated for 1h with 300μl of secondary anti-human or anti-mouse secondary antibody conjugated with AlexaFluor 488 and FITC (ThermoFisher), respectively diluted 1:100. To detect all spirochetes present in the field of view of the microscope, DNA was stained with 4',6-diamidino-2-phenylindole (DAPI) after bacterial permeabilization using cold methanol for 20 minutes. As a control to ensure that the spirochete membrane integrity was preserved during the procedure, *B*. *burgdorferi* were labeled in parallel for periplasmic flagella using a mouse anti-FlaB monoclonal antibody, provided by Dr. Errol Fikrig at Yale University, and used at 1:50 dilution for 1h to detect the lack of staining in intact, and reaction with the permeabilized bacteria. Anti-mouse secondary antibodies (1:100 dilution) conjugated with TRITC (ThermoFisher) were used for fluorescence labeling. After four washes, coverslips were mounted onto slides, and examined using Apo-Plan TIRF objective in Nikon 80i fluorescence microscope.

### Mammalian cells cultures

All cell lines were grown in an environmentally controlled incubator at 37°C in 5% CO_2_ atmosphere. African green monkey kidney epithelial Vero, C6 (rat) glioma, human umbilical vein endothelial cells (HUVEC) Ea.Hy926, and human embryonic kidney epithelial HEK293, and human epithelial HEp-2 cell lines were originally provided by Dr. John Leong at Tufts University School of Medicine. C6 glioma cell line was grown in RPMI medium supplemented with 8% FBS and Penicillin/Streptomycin (P/S) mixture, while HEK293 cells were cultivated in 1:1 mixture of Dulbecco’s Modified Eagle’s Medium (DMEM) and Ham’s F12 medium supplemented with 10% FBS and P/S mixture. Vero cells as well as HEp-2 cells were grown on RPMI medium supplemented with 10% NuSerum (ThermoFisher) and P/S mixture. Human placental fibroblast BeWo cells (ATCC^®^ CCL-98^™^) were cultured in F-12K medium supplemented with 10% FBS and P/S mixture and Ea.Hy926 cells were grown in DMEM medium with 1% HAT, 10% FBS and P/S mixture.

### Binding of radiolabeled *B*. *burgdorferi* to mammalian cells

To assess adherence, transformed *B*. *burgdorferi* B314 strains were labeled with a ^35^S methionine-cysteine mixture (Perkin-Elmer). Bacteria were harvested by centrifugation when culture density reached approximately 5×10^7^–1×10^8^ spirochetes/ml. After three five-minute washes with PBS/BSA to remove unbound label, bacterial pellets were resuspended in BSK-H medium without serum but supplemented with sterile glycerol (20% final concentration) and 1ml aliquots of labeled *B*. *burgdorferi* containing 1-2x10^8^ spirochetes per ml were stored at -80°C until use.

Binding assays were conducted using *B*. *burgdorferi* and mammalian cell lines as previously published [37, 38]. Briefly, cell cultures were plated in Nunc break-apart 96 well plates and allowed to grow for at least 24h to form a confluent monolayer. After quick thawing, B314 transformed with the shuttle vector alone and the vector containing the Tp0136 variants, respectively were centrifuged, resuspended in BSK-H and left at room temperature for two hours to recover physiologically as indicated by regaining of vigorous motility. Glucose (10mM)-HEPES (10mM, pH 7.0)-Salt (50mM) or GHS was added to obtain 2:1 ratio of GHS with bacterial suspension in BSK-H and spirochetes were incubated for an additional hour at room temperature. To determine the input bacterial count, 50μl of each culture suspension (~1x10^6^ spirochetes) were filtered through Corning Costar Spin-X centrifuge tube filters. After washing the filter with PBS and drying, radiolabel was measured using an LS 650 Multi-Purpose Scintillation Counter (Beckman Coulter). For binding determination by transfected *B*. *burgdorferi*, cell lines were washed twice with PBS, and then 50μl of each bacterial suspension containing ~1x10^6^ spirochetes were added to quadruplicate wells containing each cell line. Empty wells were also included as “no cell” controls for each treatment. After an hour of incubation at room temperature while rocking, wells were washed three times with PBS/BSA to remove unbound bacteria and plates were left to air dry. Each plate well was broken and placed in scintillation vial and 2ml Opti-Fluor O (Perkin Elmer) was added per vial. Radiolabel in each well was measured as described above. Average count obtained from four empty wells (without any bacteria or cells) was deducted from radiolabel count obtained from each well of the plate to obtain net count per well. Percent binding by the transformed B314 clone in each well was calculated using total input count for the respective clone determined as described above. Thus percent binding = (Net count per well/total input count) x100.

### Binding of radiolabeled *B*. *burgdorferi* to fibronectin and laminin

Stocks of human foreskin fibroblast fibronectin and laminin (Sigma-Aldrich) were diluted in PBS and 50μl of suspension containing 1μg of each protein was used to coat wells of a Nunc break-apart 96 well plate by incubation at 37°C for 3h. The plates were then stored at 4°C until use. After washing three times with PBS, plates were incubated at room temperature with blocking buffer containing 1% BSA in PBS for 30 minutes. After removal of blocking buffer, wells were incubated with 50μl of each labeled bacterial strain, as described above. After 1h incubation at room temperature while rocking, plates were washed and the level of bound labeled bacteria measured by scintillation counting as described above.

### Binding of fibronectin and laminin to *B*. *burgdorferi* expressing Tp0136

For examination of fibronectin and laminin binding to *B*. *burgdorferi* strains expressing allelic variants of Tp0136, bacteria were washed twice with the PBS/BSA buffer and then centrifuged on coverslips as described for IFA above. *B*. *burgdorferi* strains were incubated with 1.5μg of fibronectin or laminin suspended in 300μl PBS/BSA buffer for one hour at room temperature. After three washes for five minutes each at room temperature with PBS while shaking, 300μl of anti-fibronectin monoclonal antibody labeled with AlexaFluor 488, or anti-laminin polyclonal antibody-labeled with DyLight 550 (Thermo Fisher) diluted 1:100 in PBS/BSA were added to the respective coverslips. To detect all bacteria in each microscopic field, DNA staining was performed with DAPI after permeabilization with methanol as described for IFA above. After four washes coverslips were mounted onto slides and examined using an OLYMPUS BX61 fluorescent microscope, and images captured using the OLYMPUS CellSens Dimension 2.1 software (Olympus Corporation, Japan).

### Inhibition of binding to fibronectin, C6 and HEK293 cells by fibronectin binding peptide

FnbA-2 fibronectin-binding peptide from *Staphylococcus aureus* protein (Thermo Fisher) was used to inhibit binding of radiolabeled transformed B314 to fibronectin alone, and to HEK293 and C6 Glioma cells. Fifty microliters of PBS or DMEM (Mock for fibronectin binding and cell binding) or 200μg/ml of peptide diluted either in PBS for fibronectin-coated wells, or in DMEM for the cell lines, were added (10μg/well) and plate incubated for one hour at room temperature while rocking to allow peptide binding to immobilized fibronectin or cell monolayers. After two washings with PBS to remove unbound peptide, 50μl of radiolabeled bacteria were added to each well. After incubation for 1h while rocking at room temperature, plates were washed and dried followed by scintillation counting as described above.

### Statistical analyses

All statistical analyses were conducted using GraphPad Prism version 8.0, Software (La Jolla, CA). Data are presented as mean ± standard deviation (SD). Comparisons in binding between allelic variants of Tp0136 or among the same variant but different cell type, fibronectin and laminin was performed using unpaired student t-test for unequal variance to determine significant differences between paired groups. ANOVA was used to compare binding of both allelic variants of Tp0136 to various cell types or to fibronectin and laminin, and p values below 0.05 at 95% confidence interval to determine difference between groups were considered statistically significant.

## Results

### Comparison of Tp0136 sequences from the Nichols and SS14 strains of *T*. *pallidum*

The sequence of *T*. *pallidum* Tp0136 variants inserted into the pJSB175 plasmid and the translated *tp0136* ORFs are shown (S1 Fig). The proposed primary fibronectin-binding region of Tp0136 [27] is marked in the protein sequence that shows some variation between two alleles.

### Structural prediction and modeling of Tp0136

Predicted secondary and tertiary structural representation of the best-fit model of full Tp0136 translated ORF, based upon the highest C- and TM scores among 10 threading templates, determined by the I-TASSER server [39–41], showed β strand rich domains (S2 Fig).

### Expression and localization of Tp0136 variants on *B*. *burgdorferi* surface

**Fig 1:**
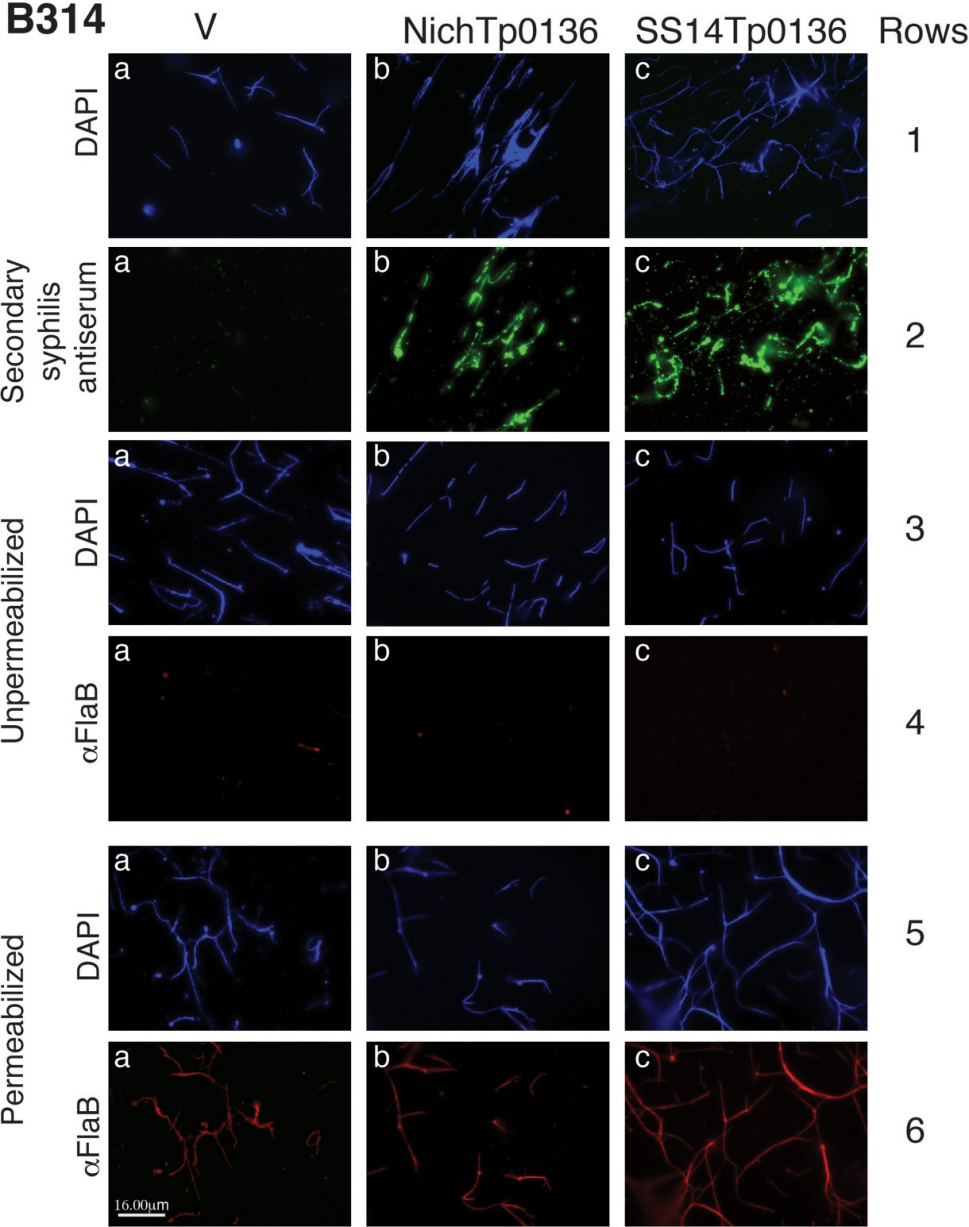
Expression of the two allelic variants of Tp0136 on *B*. *burgdorferi* strain B314 surface. Treatment of B314 strain expressing Tp0136 alleles from *T*. *pallidum* SS14 and Nichols strains with antiserum obtained from a secondary syphilis (SS) patient followed by visualization using AlexaFluor-488 conjugated anti-human IgG antibodies showed punctate green spirochetes surface staining (panels b and c in the 2^nd^ row). The SS serum did not react with other targets on the *B*. *burgdorferi* B314 control strain transformed with an empty shuttle vector (V) in the panel a in the 2^nd^ row. DNA staining by DAPI in the panels in rows 1, 3, and 5 show all bacteria present in the respective microscopic fields. A monoclonal antibody against FlaB followed by TRITC-labeled secondary anti-mouse antibodies stained spirochetes only after permeabilization with methanol (Permeabilized, panels a, b, and c in the 6^th^ row) but not intact spirochetes (Unpermeabilized, panels a, b, and c in the 4^th^ row) showing that bacterial outer membrane remained unperturbed during the IFA procedure. Bar represents 16 μm.

IFA using a secondary syphilis serum showed surface punctate labeling (green) only on B314 strains expressing the Tp0136 variants (b and c in second row of Fig 1; SS14 and Nichols) but not on B314 transformed with an empty vector used as a control (V; a in second row of Fig 1). Thus, the antiserum specifically recognized Tp0136 localized on the surface of B314 strain, and not the surface proteins of *B*. *burgdorferi* strain used in this study. Similar intensity of staining of Tp0136 from Nichols and SS14 strains of *T*. *pallidum* was detected on B314 surface (b versus c in second row of Fig 1). The absence of flagellar FlaB staining (row 4 of Fig 1) unless the spirochetes were permeabilized (row 6 of Fig 1) with methanol indicated that the integrity of the outer membrane was maintained during the assay. Similar results were also obtained in IFA using anti-Tp0136 antibodies raised in mice confirming these findings (S3 Fig); however, low titer antibodies generated in mice against Tp0136 did not label Tp0136 as intensely as the selected secondary syphilis patient serum.

### B314 expressing Tp0136 is able to bind to fibronectin-producing epithelial cell lines

**Fig 2:**
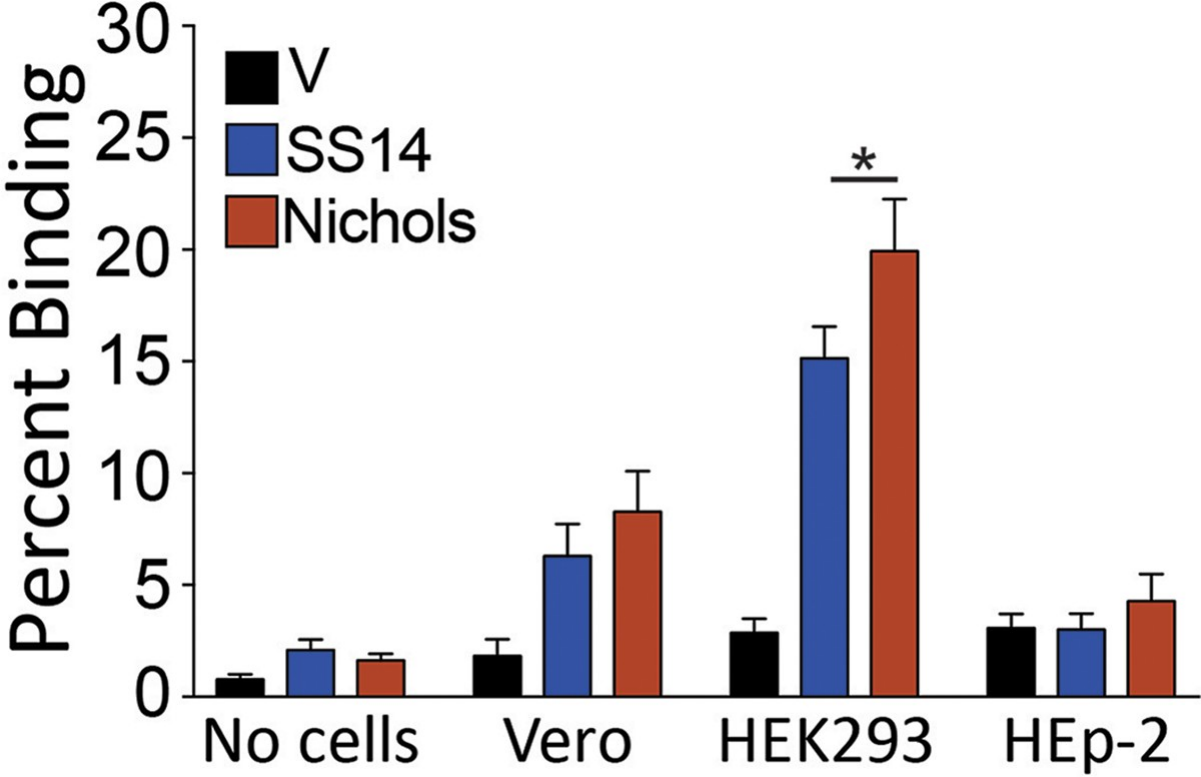
*B*. *burgdorferi* B314 strain expressing Tp0136 allelic variants bind to HEK293 more efficiently than to Vero cells but fail to adhere to HEp-2 cells. Average binding of radiolabeled *B*. *burgdorferi* B314 expressing Tp0136 variants of the SS14 and Nichols strains to Vero cells was 6% and 8%, respectively. Percent of binding mediated by Nichols strain Tp0136 to HEK293 cells was significantly higher than binding by SS14 strain Tp0136. Both variants of Tp0136 bound poorly to the HEp-2 cells, which do not produce fibronectin. Statistical analysis was conducted using a two-tailed unpaired student t test for unequal variance to determine significant difference between the paired groups and p values calculated (*p<0.05).

Binding of *T*. *pallidum* to the epithelium plays a critical role in initiating primary syphilitic lesions. Investigation of Tp0136-mediated adherence of otherwise poorly adherent *B*. *burgdorferi* B314 strain to two epithelial cell lines (Vero and HEK293) showed a significant increase in binding to Vero and HEK293 cells compared to the B314 control strain with vector alone (V; Fig 2) and was also significantly higher than the wells with no cells. Both Tp0136 variants induced similar binding levels to Vero cells (Fig 2). Binding of Tp0136-expressing B314 strains to HEK293 cells was significantly higher than to Vero cells. Although the SS14 Tp0136 protein appeared to bind to HEK293 cells slightly less efficiently compared to the Nichols strain variant (Fig 2), this difference may not be of great consequence because similar level of binding is observed by both Tp0136 alleles on all other cell lines (see below). Since Tp0136 is a fibronectin-binding protein, we further examined binding of B314 containing both alleles to control HEp-2 cell line, which is deficient in fibronectin production [42, 43]. Binding of all B314 recombinant strains to HEp-2 cells was not dissimilar from the “no cell” control wells (Fig 2) supporting that fibronectin indeed is the main Tp0136 target on the other mammalian cell lines examined here.

### Tp0136-expressing B314 strains bind to placental, glioma, and endothelial cell lines

**Fig 3:**
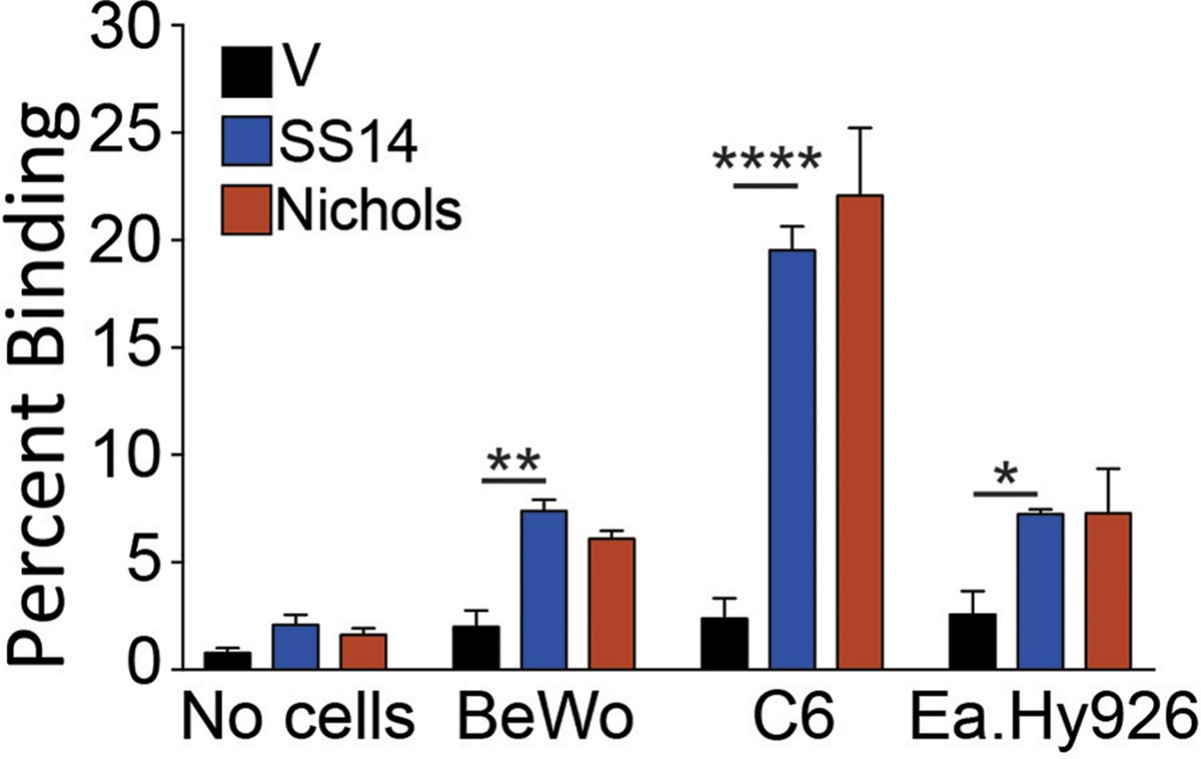
Tp0136 facilitated B314 binding to glioma, placental, and endothelial cell lines. Binding of B314 expressing Tp0136 to placental (BeWo), glioma (C6), and endothelial (Ea.Hy926) cell lines were significantly higher compared to B314 control strain. Allelic variants of the Tp0136 protein did not display significant differences in binding to any of these three cell lines. Statistical analysis was conducted using a two-tailed unpaired student t test for unequal variance to determine significant difference between the paired groups and p values calculated (*p <0.05, **p<0.01, and ***p<0.001).

Tp0136-mediated adherence of B314 surrogate strains was also analyzed using non-epithelial cells lines. We selected cell lines that are representative of tissues that *T*. *pallidum* encounters during disseminated infection. Thus, Ea.Hy926 cell line was used as a representative of endothelial cells, BeWo cells to mimic placental epithelium, and C6 glioma cells as a non-neuronal representative of central nervous system (Fig 3). Tp0136-mediated adherence of transformed B314 strains to all cell lines was significantly higher than the B314 vector control strain, with highest adherence observed on C6 glioma cells (Fig 3). However, binding of these spirochetes to placental and endothelial cells was moderate (~50% of the binding to C6 glioma cells; Fig 3). Comparable binding of both Tp0136-expressing B314 strains to the BeWo and Ea.Hy926 cells indicated that binding was not affected by Tp0136 sequence differences.

### Tp0136-expressing *B*. *burgdorferi* strains bind to human foreskin fibronectin and laminin

**Fig 4:**
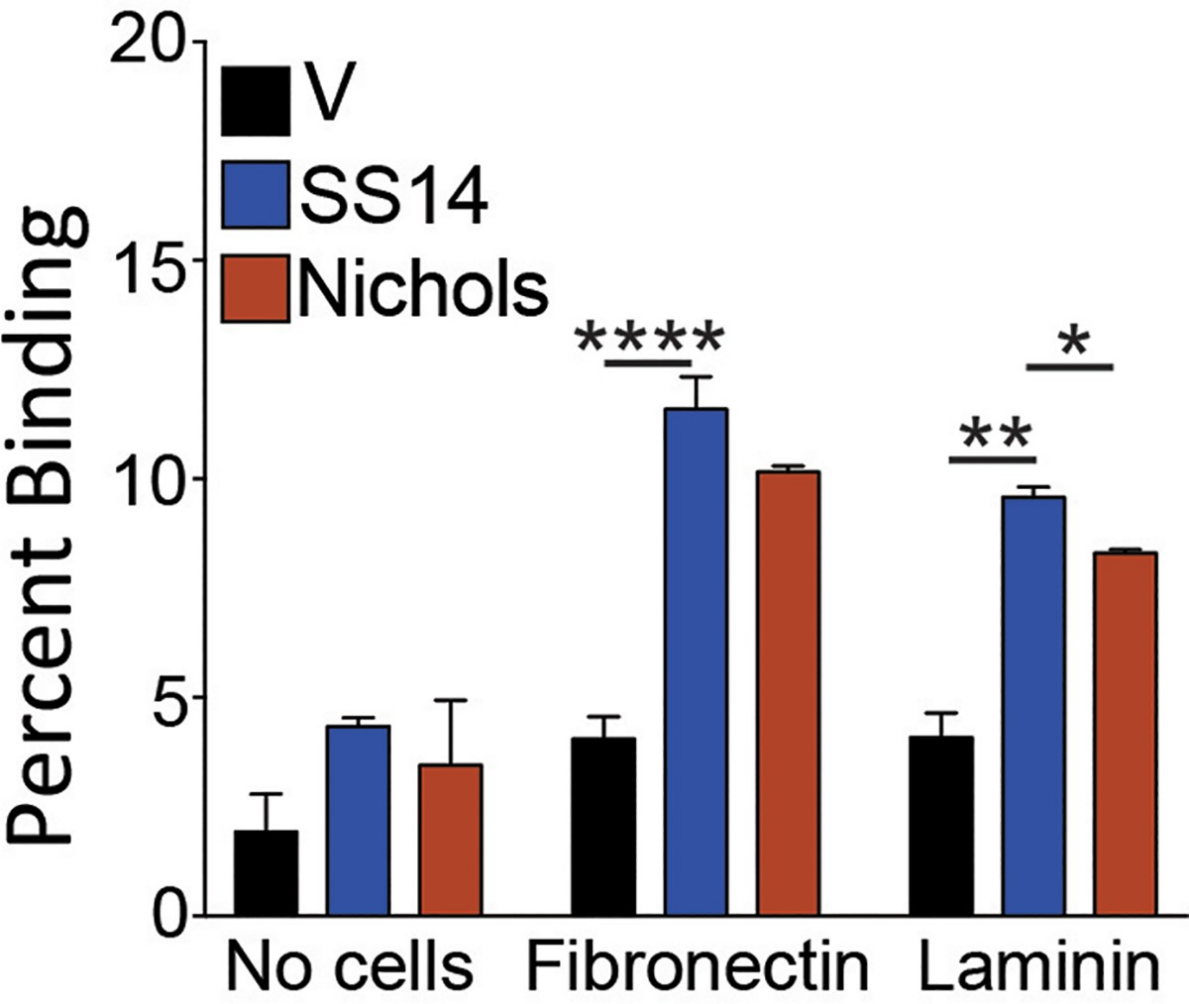
B314 expressing Tp0136 variants from Nichols and SS14 strains bind to human fibronectin and laminin. Binding of Tp0136-expressing B314 to wells coated with human tissue fibronectin was significantly higher than that of the control B314 (V) strain; however, binding facilitated by two alleles of Tp0136 was comparable to this ECM component. Binding of B314 expressing the SS14 Tp0136 to laminin was significantly higher than B314 expressing the Nichols variant. Statistical analysis was conducted using a two-tailed unpaired student t test for unequal variance to determine significant difference between the paired groups and p values calculated (*p<0.05, **p <0.01, ****p<0.0001).

**Fig 5:**
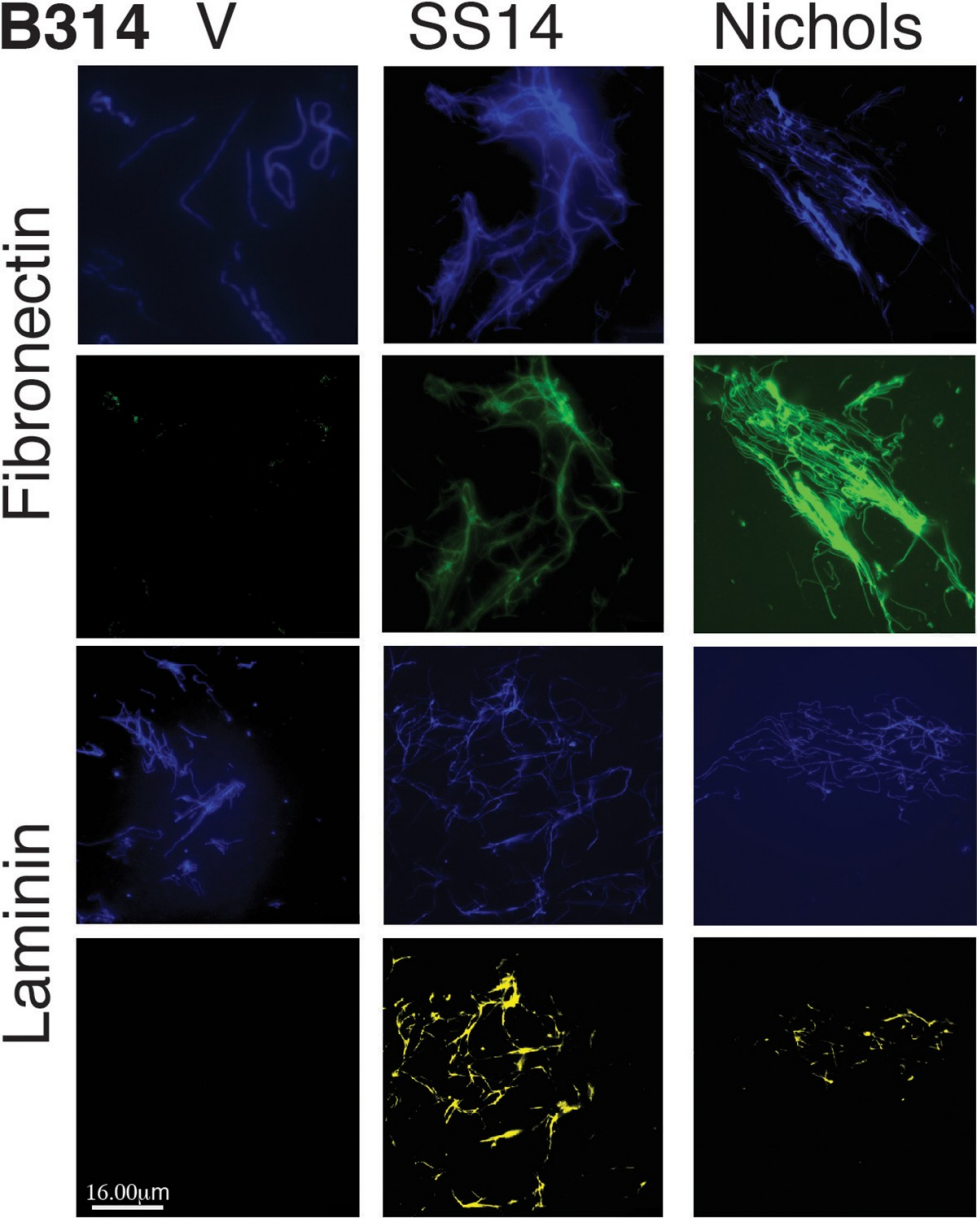
Both purified tissue fibronectin and laminin bind to Tp0136 alleles expressed on B314 strain. Spirochetes on coverglasses were incubated with purified tissue fibronectin or laminin and binding was detected using anti-fibronectin (antiFn-FITC), 2^nd^ row from top, and anti-laminin (DyLight 410) using YFP filter (antiLm-YFP) antibodies, bottom row. DAPI stained DNA in B314 transformants depict all spirochetes present in the respective microscopic fields. Top row shows all spirochetes in the field of view depicting fibronectin binding (2^nd^ row) while 3^rd^ row shows all transformed B314 in the field of view showing laminin binding (bottom row). Both allelic variants of Tp0136 showed similar efficiency in binding to fibronectin. Overall, binding of laminin to both Tp0136-expressing strains was observed with more pronounced binding to B314 expressing the SS14 Tp0136, compared to the strain expressing the Nichols variant. No laminin binding to B314(V) control was detected. Bars represent 16 μm.

Previous studies showed that Tp0136 binds to fibronectin and, less efficiently, to laminin [27, 32]. Binding of radiolabeled B314 strain expressing both variants of Tp0136 in native form to purified fibronectin was significantly higher than the control B314 strain but did not differ among Tp0136-expressing B314 strains (Fig 4). Tp0136 allele from SS14 strain mediated the higher binding to laminin than protein from Nichols strain (Fig 4) with significant differences observed among these alleles. Overall, in accordance to previous work using recombinant proteins to study adherence mechanism, laminin binding mediated by Tp0136 expressed on spirochete surface was also significantly lower than to fibronectin. We further analyzed binding of fibronectin and laminin by using a complementary IFA-based test to allow visual observation using a fluorescent microscope. B314 spirochetes expressing either of the two Tp0136 alleles were able to bind tissue fibronectin (Fig 5). B314 expressing Tp0136 from both *T*. *pallidum* strains showed moderate but detectable binding by laminin, which was not detectable on the control B314 strain (Fig 5) and was more intense on Tp0136 from the SS14 strain.

### Inhibition of binding of Tp0136-expressing B314 to fibronectin and mammalian cells by FnbA-2

**Fig 6:**
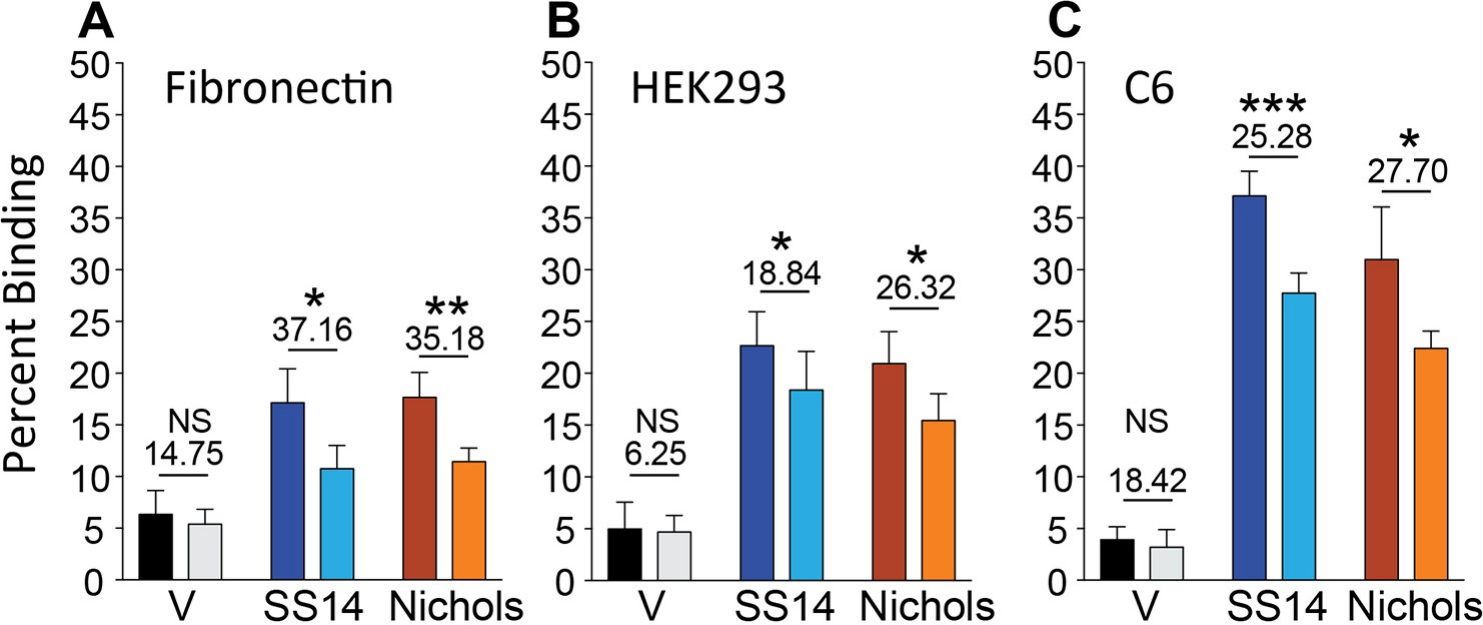
Fibronectin binding peptide FnbA-2 of *S*. *aureus* reduces attachment of Tp0136 –expressing B314 strain to purified fibronectin, and to C6 glioma and HEK293 cell lines. **(A)** Significant reduction in binding of both Tp0136 variants to immobilized tissue fibronectin was observed upon preincubation of fibronectin with FnbA-2 (light color bars) as compared to the mock treated samples (buffer only treatment, dark color bars). **(B)** Significant reduction in binding of both Tp0136 alleles expressing B314 to HEK293 cells by the FnbA-2 peptide was detected as marked by underlined numbers above the bars; however, binding mediated by two Tp0136 alleles was not significantly different on these cells (p = 0.748). **(C)** Significant inhibition of binding to C6 glioma cells by the FnbA-2 peptide was also observed. Percent inhibition of binding by FnbA-2 peptide is indicated by the underlined numbers above the bars. Statistical analysis was conducted using a two-tailed unpaired student t test for unequal variance to determine significant difference between the paired groups and p values calculated (NS-not significant, *p<0.05, **p<0.01, ***p<0.001).

We first determined if binding of Tp0136-expressing B314 strains to fibronectin can be inhibited by preincubation with the fibronectin binding peptide of *S*. *aureus* FnbA-2 protein. As expected, binding of B314 expressing different Tp0136 alleles to human fibronectin was significantly reduced when fibronectin immobilized in wells was preincubated with the FnbA-2 peptide (Fig 6A). Furthermore, to determine contribution of Tp0136-fibronectin interaction on cell binding, we preincubated cell monolayers with the fibronectin-binding peptide and quantitated B314 binding to HEK293 and C6 glioma cells by scintillation counting. We selected these cell lines because adherence to these cells was significantly higher than other cell lines we examined (Fig 2 and Fig 3). Reduction in binding of the spirochete derivatives by FnbA-2 peptide on both HEK293 and C6 glioma cells indicated that Tp0136-fibronectin interaction is involved in adherence of spirochetes to these cells. Inhibition of adherence mediated by both Tp0136 variants on HEK293 cells was significant with 18.8% and 26.3% reduction in binding for the SS14 and Nichols strain variants, respectively (Fig 6B). Comparable inhibition in binding of Tp0136 expressing B314 strains to C6 glioma cells was also observed with 25.3% and 27.70% reduction for the SS14 and Nichols strain proteins, respectively (Fig 6C). Reduction in binding of control B314(V) strain by the peptide was not statistically significant.

## Discussion

Syphilis is a chronic and systemic disease, caused by the spirochete *T*. *pallidum*. It is primarily transmitted either through sexual contact or by vertical, transplacental migration, resulting in congenital syphilis [9, 44, 45]. Due to the known limitation of working with this pathogen, investigators have heavily relied on comparative genomics to identify putative-surface-exposed virulence factors of this pathogen. [46, 47]. The newly described co-culture system for *T*. *pallidum* [8] will possibly be the gateway to genetically engineer the syphilis agent but, for the time being, manipulation of *T*. *pallidum* is still not feasible. Consequently, functional analysis of possible virulence factors using knockout *T*. *pallidum* mutants is still not an option. A recent approach adapted by several researchers to perform gain-of-function studies used *B*. *burgdorferi* as a surrogate spirochete. This system enabled investigation of the adherence mechanisms of *T*. *pallidum* to host cells, which is a critical step in the pathogenesis of this extracellular pathogen [16, 17]. This heterologous expression system also offers the advantage of eliminating interference from *T*. *pallidum* proteins with redundant functions [23, 24, 26, 27], and thus also facilitates result interpretation unambiguously. Previous recombinant protein-based assays suggested that a surface-exposed Tp0136 lipoprotein of *T*. *pallidum* that exhibits inter-strain allelic variability contributes to tissue colonization by binding to cellular and plasma fibronectin. Previous studies also showed that the TP0136 amino (N)-terminal region is primarily responsible for binding to plasma fibronectin, but that binding sites for cellular fibronectin are also present in the protein's central and carboxyl (C)-terminal regions [27, 32].

The current study was undertaken to confirm Tp0136 surface exposure when the protein is expressed in a poorly adherent *B*. *burgdorferi* surrogate strain and to further confirm its function in its close to normal conformation. The possibility that lipoproteins gain surface-exposure in *T*. *pallidum* is in fact a fairly new finding that is still amply debated, even though evidence toward that direction is mounting [16, 48]. Bacterial lipoproteins are expected to contain amino-terminal cysteine (+1 position of the mature protein) with lipid modification. Based upon sequence analysis of Tp0136, it is difficult to determine identity of the first cysteine of the mature lipoprotein with certainty. According to SpLip predictions, spirochete lipobox consists of five amino acids at the carboxyl-terminal end of the signal peptide that is cleaved by a signal peptidase II. Its characteristics are that Ala, Gly, Ser, Asn or Cys are allowed at the -1 position, while Leu, Ile, Val and Phe should be present at positions -3 and/or -4. Charged amino acids like Lys, Arg, His, Asp, and Glu are prohibited in the lipobox [29]. This suggests that the likely lipobox for the mature Tp0136 is LLTT**C** (AA 30–34) is the target of signal peptidase II, although this model does not completely fits the above criteria for the -1 position (S1 Fig). Predicted model of Tp0136 using I-TASSER server showed primarily β-strand rich domains (S2 Fig). Crystal structures of other lipoproteins of spirochetes with several β-strands rich domains have also been shown previously [49–52].

We demonstrated that the expression of two distinct allelic variants of Tp0136 on the surface of *B*. *burgdorferi* B314 strain is similar (Fig 1). Moreover, both secondary syphilis patient and mouse sera antibodies recognized Tp0136 allelic isoforms and did not label *B*. *burgdorferi* surface in a non-specific way. Also, as already known [16, 17, 48], our study shows that Tp0136 expressed on the surface of the surrogate spirochete increases binding to tissue fibronectin more than to laminin (Figs 4 and 5). Collectively, these results indicate that when *B*. *burgdorferi* is used as a heterologous surrogate system to express surface proteins of *T*. *pallidum*, it correctly sorts them to their natural cellular compartment, allowing gain-of-function approach feasibility. Infectious *B*. *burgdorferi* possesses at least two fibronectin-binding proteins, BBK32 and BB0347. An endogenous plasmid-borne gene encodes BBK32 while the *bb0347* gene is located on the chromosome [42, 53–55]. Although B314 strain has lost the endogenous plasmid carrying the *bbk32* gene [56], binding of the control B314 strain (transformed with the empty shuttle vector) can be attributed to the low level expression of BB0347 [57]. With such premises, the differences seen when using Tp0136-expressing strains can be attributed primarily to the fibronectin/laminin-binding protein of the syphilis spirochete. The Nichols and SS14 variants (S1 Fig) of Tp0136 show nearly 90% identity and 92% similarity, with differences spread almost evenly throughout the length of the protein. According to the results of the binding experiments, these differences do not appear to play a significant role in determining Tp0136 binding efficiency to purified fibronectin (Figs 4 and 5), and to mammalian cells (Figs 2 and 3).

High level of binding to HEK293 cells and not to Vero cells, which are also epithelial cells, might simply reflect differences in the levels of fibronectin and/or laminin expression in these cell lines. Nonetheless, the lack of binding to the HEp-2 cells, which is deficient in producing fibronectin [42, 43], is a clear indicator of the importance of fibronectin-Tp0136 interaction for *T*. *pallidum* adhesion to host cell components (Fig 2). The difference in binding mediated by SS14 strain Tp0136 compared to that of Nichols strain Tp0136 to HEK293 cells, and better recognition of laminin by SS14 Tp0136 (Figs 4 and 5), suggest that allelic differences could play a moderate role in attachment to different cells during infection, as mediated by differential levels of expression of the receptor(s). Relatively lower levels of adherence to human placental and endothelial cells (Fig 3) compared to HEK 293 cells further supports the premise that Tp0136 of *T*. *pallidum* could play a role in differential cell binding-mediated colonization of different tissues during infection, which could be potentially again be attributed to the levels of fibronectin and laminin present on the specific host cell surface. High levels of binding of B314 expressing different Tp0136 variants to C6 glioma cells (Fig 3) suggest importance of this protein in tropism of *T*. *pallidum* to cells associated with nervous system. Overall, our results indicate that Tp0136 may contribute to colonization of tissues during infection by recognition of these target proteins.

Although inhibition of adherence to purified receptors and host cells by the specific antibodies have been used for different pathogens, we decided not to use inhibition of bacterial binding by the specific antibodies. This is because in our (N.P.) extensive experience in studying adherence of difference spirochetes to host cells [37, 38, 58–61], we have found that; (a) in many cases antibodies are bactericidal or affect integrity of the spirochetes [38, 62–67], (b) antibodies can facilitate formation of spirochete clumps that could interfere in proper binding of bacteria, and (c) antibodies can exaggerate reduction in binding by steric inhibition due to their large size. The last two points are also applicable when preincubation of the spirochetes with purified fibronectin is used for inhibition of binding of bacteria to the host cells. Therefore, we decided to use the *S*. *aureus* FnbA-2 peptide as inhibitor of adherence. We observed a significant inhibition in attachment of Tp0136-expressing B314 strains to fibronectin, and HEK293 and C6 glioma cells when they were preincubated with FnbA-2 (Fig 6). Interestingly, fibronectin binding to Tp0136 also shows significant staining in IFA (Fig 5) suggesting its correlation with the ability of FnbA-2 peptide to block interaction of Tp0136 with fibronectin present on the host cells.

Tp0136 allelic variability could have evolved as a way to generate surface antigenic diversity among *T*. *pallidum* strains as a general mechanism to facilitate immune evasion while maintaining its basic function of adherence to the host cells. However, allelic variants of Tp0136 do not seem to be drive significant differences in binding to cells, albeit both show specific affinity for some cell types. In summary, our results show that Tp0136 mediates differential adherence specificity to cell lines derived from different tissues, likely dependent on the level of fibronectin and/or laminin produced by these cells, and reiterate the notion of a role for this virulence factor in helping the syphilis agent to colonize various tissues.

## Supporting information

S1 Fig Comparison of the sequence from *Treponema pallidum* SS14 and Nichols strain. **(A)** DNA insert containing *tp0136* gene (blue) and upstream and downstream sequence (orange) in *Borrelia burgdorferi* shuttle vector, and **(B)** Tp0136 Open Reading Frame (ORF) with marked early cysteine residues (red), lipobox (bold blue underlined with putative first cysteine residue of mature lipoprotein marked red) and putative fibronectin binding region (blue).
(DOCX)

S2 Fig Structural prediction of Tp0136 protein of Nichols and SS14 strains of *T*. *pallidum* using I-TASSER server. **(A).** Amino Acid sequence and predicted secondary structure of Tp0136 proteins determined by the I-TASSER server. The signal peptide sequence is in green and the putative first cysteine residue of the mature Tp0136 lipoprotein is in purple and underlined. **(B)** Three-dimensional representation of the best-fit model of Tp0136 proteins (Nichols and SS14 strains) based upon the highest C, and TM scores using 10 threading templates. White arrow marks the helical domain of the signal peptide of Tp0136 while gray marked region depicts predicted fibronectin binding domains.
(TIF)

S3 Fig IFA depicting surface labeling of Tp0136 on B314 surface using anti-Tp0136 mouse serum. Low antibody titer polyclonal antibodies generated against recombinant Tp0136 in Balb/c mice did not label control B314 containing the empty vector, i.e., B314(V), and weakly reacted with the SS14 and Nichols Tp0136 expressed on B314 strain surface (bottom row). Anti-mouse FITC-conjugated secondary antibodies marked the spirochetes green. All spirochetes present in the microscopic fields, with DNA stained with DAPI, are shown in the top row. Bar represents 16 μm.
(TIF)
