# Supplementary material for: Analysis of host cell binding specificity mediated by the Tp0136 adhesin of the syphilis agent Treponema pallidum subsp. pallidum
Source: PLoS Negl Trop Dis. 2019 May 9;13(5):e0007401. doi: 10.1371/journal.pntd.0007401 (PMC6529012; doi:10.1371/journal.pntd.0007401)
Supplement: S1 Fig — (A) DNA insert containing tp0136 gene (blue) and upstream and downstream sequence (orange) in Borrelia burgdorferi shuttle vector, and (B) Tp0136 Open Reading Frame (ORF) with marked early cysteine residues (red), lipobox (bold blue underlined with putative first cysteine residue of mature lipoprotein marked red) and putative fibronectin binding region (blue). (DOCX) [file pntd.0007401.s001.docx]

**S1 Figure**

**A.**

Nichols 1 TCTATTACGAGAAGGAGCGGCTGTGTTCCTACCTTGCCGTTCCTGTAACGCTGAATGCAAGTTACACGCTCGCCTTCTGG 80

SS14 1 TCTATTACGAGAAGGAGCGGCTGTGTTCCTACCTTGCCGTTCCTGTAACGCTGAATGCAAGTTACACGCTCGTCTTCTGG 80

************************************************************************ *******

Nichols 81 CGCATCCGTCTGCCGCTGACGGTGGGGGCCGGCTTCAGCTACCAGCACTACTACACGTCTACGTACTACGGTCTTTTGCT 160

SS14 81 CGCATCCGTCTGCCGCTGACGGTGGGGGCCGGCTTCAGCCACCAGCACTACTACACGTCTACGTACTACGGTCTTTTGCT 160

*************************************** ****************************************

Nichols 161 CAAAGCAGGGGCCGGCTGTTACCTTCAGCTGACAGAGCGCTGGTCCTTGGGTGCCAGTGCTGCATATAGCGGCGTACCCC 240

SS14 161 CAAAGCAGGGGCCGGCTGTTACCTTCAGCTGACAGAGCGCTGGTCCTTGGGTGCCAGTGCTGCATATAGCGGCGTACCCC 240

********************************************************************************

Nichols 241 GGTCGTGCGAAAAAGTGGTTGAAGAAGAGAGAGAGCAGACTAACACGCGCACTGCGCAGTTTGTCGCCGCCGGGGTAGAT 320

SS14 241 GGTCGTGCGAAAAAGTGGTTGAAGAAGAGAGAGAGCAGACTAACACGCGCACTGCGCAGTTTGTCACCGCCGGGGTAGAT 320

***************************************************************** **************

Nichols 321 ATCCGCTATCACCTCTGACGCGCGCATCCGCCGAGTCCTGGGCATGCCGCCGAATGCAGTACATCGTGTTGCGCGCCGCG 400

SS14 321 ATCCGCTATCACCTCTGACGCGCGCATCCGCCGAGTCCTGGGCATGCCGCCGAATGCAGTACATCGTGTTGCGCGCCGCG 400

********************************************************************************

Nichols 401 CAGTGTCGGGGTATCACGGAAGGCGGTACAGGCACTGCGTGCACGGCGCGCTTGTGCGTCGCGCGCGGTCGCATACCAAA 480

SS14 401 CAGTGTCGGGGTATCATGGAAGGCGGTACAGGCACTGCGTGCACGGCGCGCTTGTGCGTCGCGCGCGGTCGCATACCAAA 480

**************** ***************************************************************

Nichols 481 ACAATACGCAGGGCGCTTAGCTCAGCGGGCAGAGCGCTTGGTTTACACCCAAGAGGTCAGCGGTTCAAACCCGTTAGCGC 560

SS14 481 ACAATACGCAGAGCGCTTAGCTCAGCGGGCAGAGCGCTTGGTTTACACCCAAGAGGTCAGCGGTTCAAACCCGTTAGCGC 560

*********** ********************************************************************

Nichols 561 CCAACATGCAGCCGCGTTTCTGCTATCGGATAAAGGACGACTGGGCTGGCAGCGGGTGTGGGTTCCCACCTCCTGTTCGT 640

SS14 561 CCAACATGCAGCCGCGTTTCTGCTATCGGATAAAGGACGACTGGGCTGGCAGCGGGTGTGGGTTCCCACCTCCTGTTCGT 640

********************************************************************************

Nichols 641 GTCTTTTCAGGGTGTGTGCGCGTTCCGAGAAGAGGGCGTTTTGTGT**GTG**GGGAGGAGTACGATGGATACGCAGTATATGA 720

SS14 641 GTCTTTTCAGGGTGTGTGCGCGTTCCGAGAAGAGGGCGTTTTGTGT**GTG**GGGAGGAGTACGATGGATACGCAGTATATGA 720

********************************************************************************

Nichols 721 GGCGCCGGGTGTGCACGGTGGTGCGCGCGGTGGTGTGTCTACTCAGCACGAGTTTGCTGACCACGTGCGATTTCACTGGC 800

SS14 721 GGCGCCGGGTGTGCACGGTGGCGCGCGCGGTGGTGTGTCCACTCAGCACGAGTTTGCTGACCACGTGCGATTTCACTGGC 800

********************* ***************** ****************************************

Nichols 801 ATCTTTGCGGCAATTCAGTCGGAAGTGCCCATTAAAACGCCGTCCATCCCGGGGGCGATTTATGGCCTGGTCAAGGCCGG 880

SS14 801 ATCTTTGCGGCAATTCAGTCGGAAGTGCCCATTAAAACGCCGTCCATCCCGGGGGCGATTTATGGCCTGGTCAAGGCCGG 880

********************************************************************************

Nichols 881 GAGCAAGCTCTACGCCACCAACGGCCGGCTTTGGGAAAAGGAGCTGAACGGCACTGGGTCGTGGCAGAAAGTGTCTTCCT 960

SS14 881 GAGCAAGCTCTACGCCACCAACGGCCGGCTTTGGGAAAAGGAGCTGAACGGCACTGAGTCGTGGCAGAAAGTGTCTTCCT 960

******************************************************** ***********************

Nichols 961 CGTCCGTTCCCACTGACTCGGATAAAAAGGTTATGAGCATTGCCACCGACGGGAACACGTTCGTCCTCGCCTGCGTGCCT 1040

SS14 961 CGTCCGTTCCCACTGACTCGGATAAAAAGGTTATGAGCATTGCCACCGACGGGACCAATTTCGTCCTCGCCTGCGTGCCT 1040

****************************************************** ** *********************

Nichols 1041 GGCACGGGCGTCTACAAACATTGCGTAAATGGCGCGGGCAGCTCAAGCACCGGCACAACGGCAAGCCCCTCGACTGAAAC 1120

SS14 1041 GGCACGGGCGTTTACAAACACTGCGTAAATGGCGCGGTCGGC---AGCAGCAGCACAGCGGCAAGCGGCTCGACTGAAAC 1117

*********** ******** **************** * ** **** * ***** ******** ************

Nichols 1121 CTGCTCGCAGCATGCGACGCTCGTGGGGGGAACGTCCAAGCCCTTCTGGCTCGTGCCGGGAGGCACGGGGAATAATGGGA 1200

SS14 1118 CTGCTCGAATCATGCGACGCTCGTGGGGGGAACGTCCACGCCCTTCTGGATCGTGCCGGGAGGCACGGGGAGTAATGGGA 1197

******* * **************************** ********** ********************* ********

Nichols 1201 ACTGCGGTTGCGGGGGAGGGGGGGGTGGCTCCTCCTCGAGTAGCAGCTCGTGCATTCACATCTGGCTCGTGCCGGGAGGC 1280

SS14 1198 ACTGCGGTTGCGGGGCAGGGGGGGGTGGCTCCTCCTCCAGTAGCAGCTCGTGCATTCACATCTGGCTCGTGCCGGCAGGC 1277

*************** ********************* ************************************* ****

Nichols 1281 ACGGGGAATAATGGGAACTGCGGTTGCGGGGGAGGGGGGGGTGGCTCCTCCTCGAGTAGCAGCTCGTGCATTCACATTAA 1360

SS14 1278 ACGGGGAGTAATGGGAACTGCGGTTGCGGGGCAGGGGGGGGTGGCTCCTCCTCCAGTAGCAGCTCGTGCATTCACATTAA 1357

******* *********************** ********************* **************************

Nichols 1361 GGTAGAAAACACGGACGAACAGTTTCTCGATATGGGTGAGGGGTACGTGGTGACCACCAAGCACCTCTACACCAAAAACG 1440

SS14 1358 GAAAGAAGACACGGGCGAACAGTTTCTCGATAGGGGTGAGGGGTACGTGGTGACCACCAAGCACCTCTACACCAAAAACG 1437

* **** ****** ***************** ***********************************************

Nichols 1441 GCTCGTCCAGCGCGGGACCGGCGCAGTGTCCCGGTGGCGGTGGCGGCGGAGGCAGCAGCGGGGGTGGGGGTTCCTCGGAG 1520

SS14 1438 GCTCGTCCAGCGCGGGACCGGCGCCGTGTCCCGGT------GGCGGCGGAGGCAGCAGCGGGGGTGGGGGTTCCTCGCAG 1511

************************ ********** ************************************ **

Nichols 1521 TACACCAAAGCTTCCTGTTCCTTTTCCACGCCCATTCTGGCAAGCGTCAGCGACGGGTGCTATCACTACATTCTCACCAA 1600

SS14 1512 TACACCAAAGATTCCTGTTTCTTTTCCACGCCCATTCTGGCAAGCGTCAGCGACGGGTGCTATCACTACATTCTCACCAA 1591

********** ******** ************************************************************

Nichols 1601 AGAAAAAGTGTACTGCAGAAAGCAGGACACCGCTTCCTCCGCTGCGTCGTCACCAGCCCAGTGTCCCTCTTCCCCTTCTT 1680

SS14 1592 AGAAAAAGTGTACTGCAGAAAGCAGAATACCGCTTCCTCCGCTGCGTCGTCACCAGCCTCGTGTCCCTCTTCCCCTTCTT 1671

************************* * ****************************** ********************

Nichols 1681 CTTCTTCCTCCTCCTCGACGAATGCGGGATGCGAGGTGGCGCACGGGGTGGACGACCCGCTGTGTCTTGCGATTTTTAAA 1760

SS14 1672 CTTCTTCCTCCTCCTCGACGAATGCGGGATGCGAGGTGGAGCACGGGGTGGACGACCCGCTGTGTCTTGCGATTTTTAAA 1751

*************************************** ****************************************

Nichols 1761 CACAACGGCTGCGAATACTTGCTCATCGGCGGCAGTCGGGGCTACGGGGAAATAAAGCTGGAAGCGAACTCCAGCGGTAC 1840

SS14 1752 CACAACGGCTGCGAATACTTGCTCATCGGCGGCAGTCGGGGCTACGGGGAAATAAAGCTGGAAGCGAGCTCCAGCGGTAC 1831

******************************************************************* ************

Nichols 1841 GAACGGCACCTGCATGCGATTGAAAGAGAGCAATGTGCACAAGAGTCCGGGCCAGTGGGGCGAGTCGAGCCCCACGCCCA 1920

SS14 1832 GAACGGCACCTGCATGCGATTGAAAGAGAGCAATGTGCACAAGAGTCCGGACCAGTGGGACGAGTCGAGCCCCACGCCCA 1911

************************************************** ******** ********************

Nichols 1921 AAGCGAGCGCCGAGCAGTATCGGGGCACGGTCGGTCGGTTTGCCGTGCAGAAAATCTACGTAGTTGAAAAAAATGGCGGT 2000

SS14 1912 AAGCGAGCGCCGAGCAGTATCGGGGCACGGTCGGTCGGTTTGCCGTGCAGAAAATCTACGTAGTTGAAAAAAATGGCGGT 1991

********************************************************************************

Nichols 2001 GGGAACGGTGTCGCCGCGGGTGGGGCGGGCTGTCCTGCAAACGCCAGCAGTTCCAGCGGAGGGACCAGCAGCACGCAGCG 2080

SS14 1992 GGGAACGGTGTCGCCGCGGGTGGGGCGGGCTGTCCTGCAAGCGCCAGCAGTACCAACGGAACGGCCGGCAGCACGCAGCG 2071

**************************************** ********** *** **** * ** *************

Nichols 2081 TCCAGACCTCTACGCCGCAGTGGGGGAGTCGAGCGACACCTACACGGGGCTGTGGAAGTTTGACACCACCACGTGCTCCT 2160

SS14 2072 TCCAGACCTCTACGCCGCAGTGGGGGAGTCGAGCGACTCGTACACGGGGCTGTGGAAGTTTGACACCACCACGTGCTCCT 2151

************************************* * ****************************************

Nichols 2161 GGAACCGCGAG**TAA**CGGGGTGTTTTCTTTCTTCATCTGAGCAAGCCCTACCCCCAGCCATTTACTTCCCCCTGCTGCTCC 2240

SS14 2152 GGAACCGCGAG**TAA**CGGGGTGTTTTCTTTCTTCATCTGAGCAAGCCCTACCCCCAGCCATTTACTTCCCCCTGCTGCTCC 2231

********************************************************************************

Nichols 2241 TGCTACGAGGTGGAGCAAGGAATATTGTGGGTATTACGAGTGTGGGGTAGTGGTCAGTCCGTTAGAGAAGGTGGAGATTC 2320

SS14 2232 TGGTACGAAGTGGAGCAAGGAATATTGTGGGTATTACGAGTGTGGGGTAG-GGTCAGTCCGTTAGAGAAGGTGGAGATTC 2310

** ***** ***************************************** *****************************

Nichols 2321 GGCTGAGCTGGGAGCAAGGCAAGCTACAAGAGAACAGCAATGTAGTGATAGAGAAGAACGTGACGGAGCGTTGTCAATTC 2400

SS14 2311 GGCTGAGCTGGGAGCAAGGCAAGCTACAAGAGAACAGCAATGTAGTGATAGAGAAGAACGTGACGGAGCGTTGTCAATTC 2390

********************************************************************************

Nichols 2401 GTAGGGACTGTCCCTCGGTGGGTGTGCTCACGCGAGGGACAGGGGTTCGTCCGAAGCGGGTTGCTTAGAAATATCCACTA 2480

SS14 2391 GTAGGGACTGTCCCTCGGTGGGTGTGCTCACGCGAGGGACAGGGGTTCGTCCGAAGCGGGTTGCTTAGAAATATCCACTA 2470

********************************************************************************

Nichols 2481 AAAAGTGCGAAATGTTACCAACTGCCGAGAAAACTGGGATGAACACAATGCCCCCCTCTTGCTCCACGGGCGTGTGCGTC 2560

SS14 2471 AAAAGTGCGAAATGTTACCAACTGCCGAGAAAACTGGGATGAACACAATGCCCCCCTCTTGCTCCACGGGCGTGTGCGTC 2550

********************************************************************************

Nichols 2561 TTTTCCGCTTCTTGGACTGCGCGTGTACTGTCTGTTGCCGGAAAGATATGTTCAATCGTGAGGGTTTTGTCTGC 2634

SS14 2551 TTTTCCGCTTCTTGGACTGCGCGTGTACTGTCTGTTGCCGGAAAGATATGTTCAATCGTGAGGGTTTTGTCTGC 2624

**************************************************************************

**B.**

Nichols 1 VGRSTMDTQYMRRRV**C**TVVRAVV**C**LLSTS**LLTTC**DFTGIFAAIQSEVPIKTPSIPGAIYGLVKAGSKLYATNGRLWEKEL 80

SS14 1 VGRSTMDTQYMRRRV**C**TVARAVV**C**PLSTS**LLTTC**DFTGIFAAIQSEVPIKTPSIPGAIYGLVKAGSKLYATNGRLWEKEL 80

****************** ***** *******************************************************

Nichols 81 NGTGSWQKVSSSSVPTDSDKKVMSIATDGNTFVLACVPGTGVYKHCVNGAGSSSTGTTASPSTETCSQHATLVGGTSKPF 160

SS14 81 NGTESWQKVSSSSVPTDSDKKVMSIATDGTNFVLACVPGTGVYKHCVNGAVGSSS-TAASGSTETCSNHATLVGGTSTPF 159

*** *************************++******************* **+ *+** ******+********* **

Nichols 161 WLVPGGTGNNGNCGCGGGGGGSSSSSSSCIHIWLVPGGTGNNGNCGCGGGGGGSSSSSSSCIHIKVENTDEQFLDMGEGY 240

SS14 160 WIVPGGTGSNGNCGCGAGGGGSSSSSSSCIHIWLVPAGTGSNGNCGCGAGGGGSSSSSSSCIHIKKEDTGEQFLDRGEGY 239

*+****** ******* ******************* *** ******* **************** * * ***** ****

Nichols 241 VVTTKHLYTKNGSSSAGPAQCPGGGGGGGSSGGGGSSEYTKASCSFSTPILASVSDGCYHYILTKEKVYCRKQDTASSAA 320

SS14 240 VVTTKHLYTKNGSSSAGPAPCPGGGGG--SSGGGGSSQYTKDSCFFSTPILASVSDGCYHYILTKEKVYCRKQNTASSAA 317

******************* ******* ********+*** ** **************************** ******

Nichols 321 SSPAQCPSSPSSSSSSSTNAGCEVAHGVDDPLCLAIFKHNGCEYLLIGGSRGYGEIKLEANSSGTNGTCMRLKESNVHKS 400

SS14 318 SSPASCPSSPSSSSSSSTNAGCEVEHGVDDPLCLAIFKHNGCEYLLIGGSRGYGEIKLEASSSGTNGTCMRLKESNVHKS 397

**** ******************* *********************************** *******************

Nichols 401 PGQWGESSPTPKASAEQYRGTVGRFAVQKIYVVEKNGGGNGVAAGGAGCPANASSSSGGTSSTQRPDLYAAVGESSDTYT 480

SS14 398 PDQWDESSPTPKASAEQYRGTVGRFAVQKIYVVEKNGGGNGVAAGGAGCPASASSTNGTAGSTQRPDLYAAVGESSDSYT 477

* ** ********************************************** ***+ * + ****************+**

Nichols 481 GLWKFDTTTCSWNRE* 496

SS14 478 GLWKFDTTTCSWNRE* 493

****************
