# Supplementary figures and images for: Analysis of host cell binding specificity mediated by the Tp0136 adhesin of the syphilis agent Treponema pallidum subsp. pallidum
Source: PLoS Negl Trop Dis. 2019 May 9;13(5):e0007401. doi: 10.1371/journal.pntd.0007401 (PMC6529012; doi:10.1371/journal.pntd.0007401)

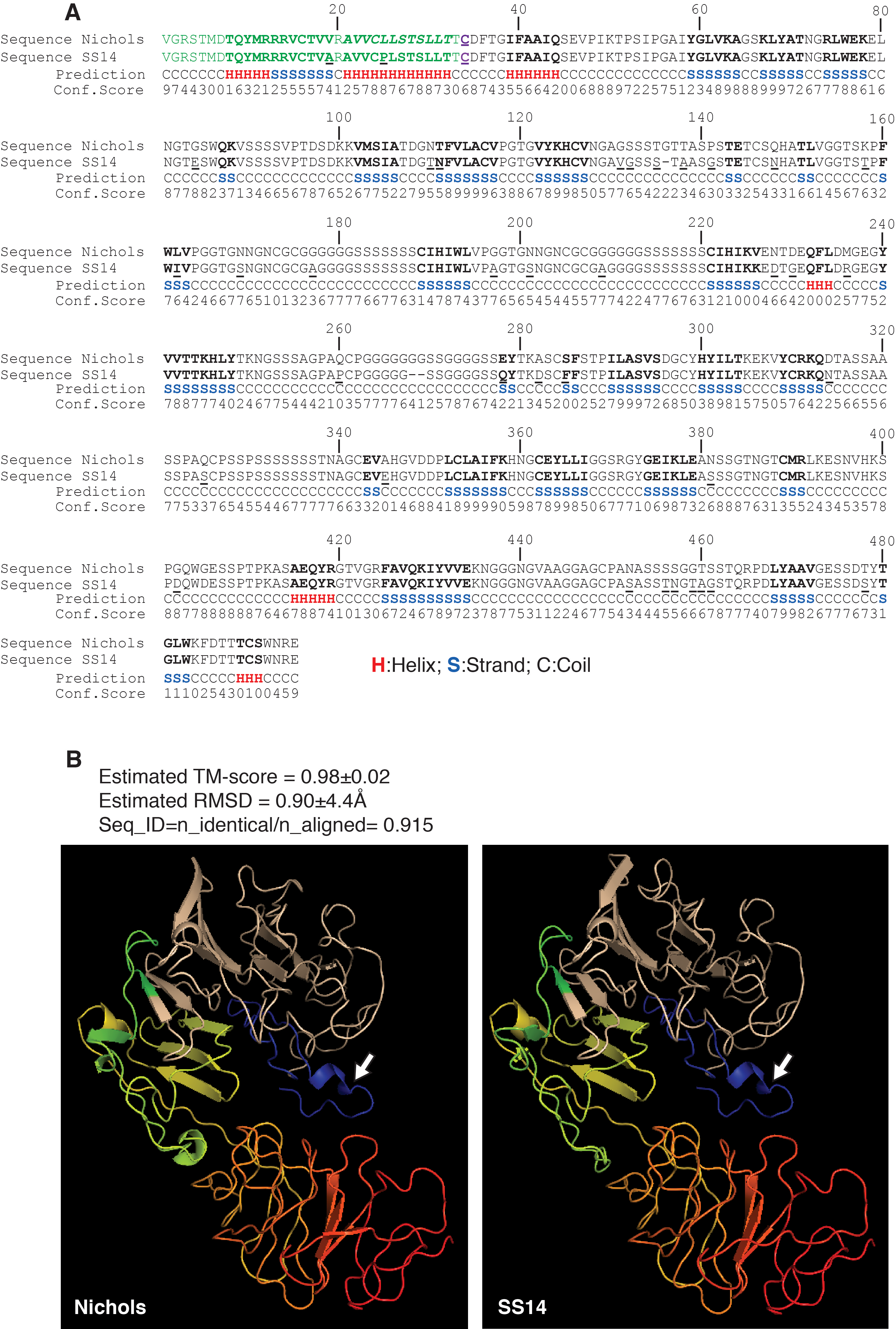

Supplement: S2 Fig — (A). Amino Acid sequence and predicted secondary structure of Tp0136 proteins determined by the I-TASSER server. The signal peptide sequence is in green and the putative first cysteine residue of the mature Tp0136 lipoprotein is in purple and underlined. (B) Three-dimensional representation of the best-fit model of Tp0136 proteins (Nichols and SS14 strains) based upon the highest C, and TM scores using 10 threading templates. White arrow marks the helical domain of the signal peptide of Tp0136 while gray marked region depicts predicted fibronectin binding domains. (TIF) [file pntd.0007401.s002.tif]

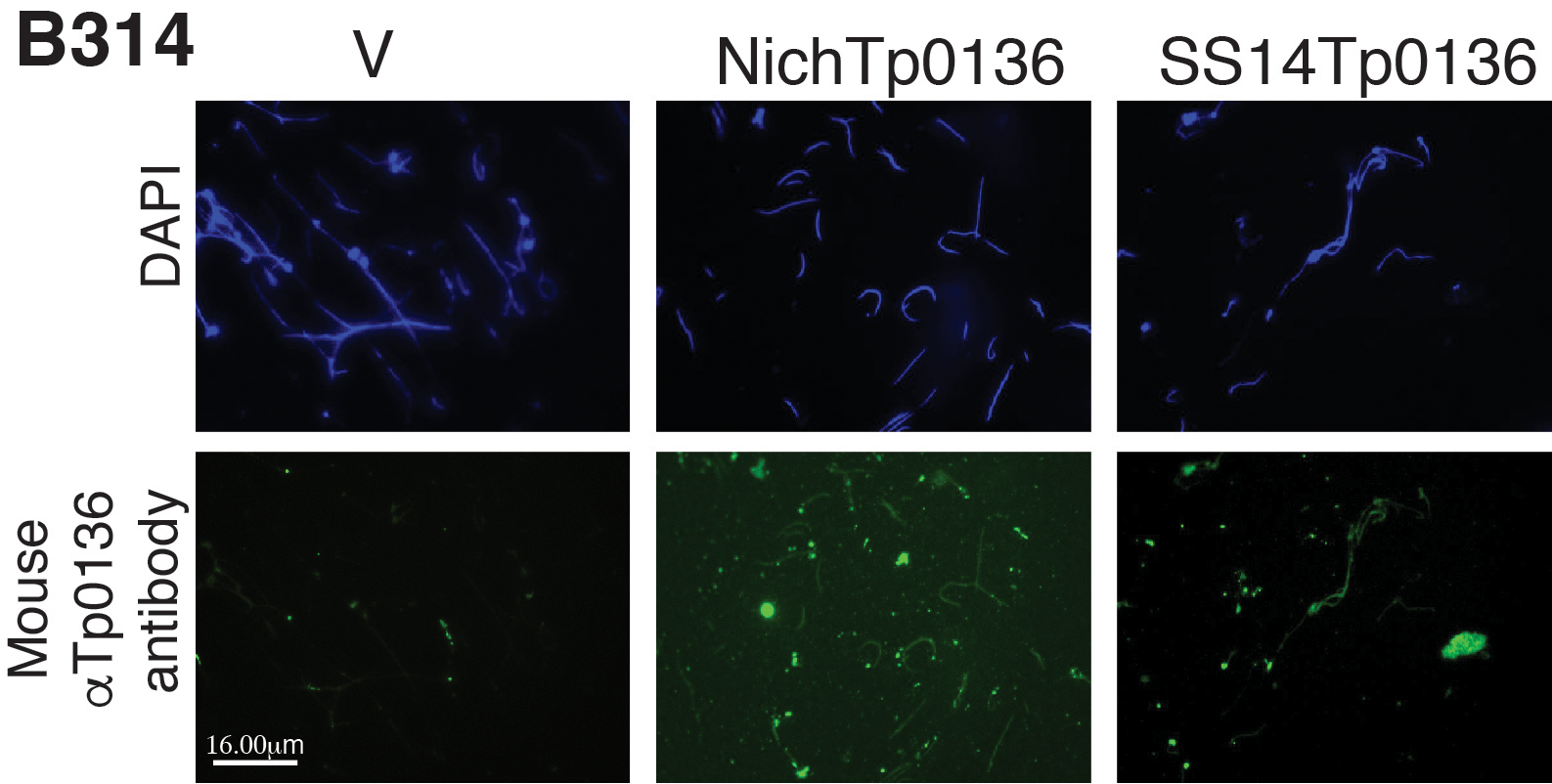

Supplement: S3 Fig — Low antibody titer polyclonal antibodies generated against recombinant Tp0136 in Balb/c mice did not label control B314 containing the empty vector, i.e., B314(V), and weakly reacted with the SS14 and Nichols Tp0136 expressed on B314 strain surface (bottom row). Anti-mouse FITC-conjugated secondary antibodies marked the spirochetes green. All spirochetes present in the microscopic fields, with DNA stained with DAPI, are shown in the top row. Bar represents 16 μm. (TIF) [file pntd.0007401.s003.tif]
